# American Medical Association

**Published:** 1848-05

**Authors:** 


*American Medical Association.*—The American Medical Association met at Baltimore on the 2d inst., and closed its session on the 4th inst. The number of delegates present was 277.

The officers elected for the ensuing year are as follows:—

President, Dr. A. H. Stevens, of New-York; Vice Presidents, Drs. Samuel Jackson, of Philadelphia; John C. Warren, of Boston; Paul T. Eve, of Georgia, and Wm. M. Awl, of Ohio.

Secretaries, Drs. Alfred Stille, of Philadelphia, and H. J. Bowditch, of Boston.

Treasurer, Dr. Isaac Hays, of Philadelphia.

The next annual meeting is to be held at Boston, Massachusetts.

We expect soon to receive full reports of the proceedings, &c., and shall lay them before our readers in our next number.

